# Heavy precipitation, drinking water source, and acute gastrointestinal illness in Philadelphia, 2015-2017

**DOI:** 10.1371/journal.pone.0229258

**Published:** 2020-02-24

**Authors:** Anneclaire J. De Roos, Michelle C. Kondo, Lucy F. Robinson, Arjita Rai, Michael Ryan, Charles N. Haas, José Lojo, Jerald A. Fagliano

**Affiliations:** 1 Department of Environmental and Occupational Health, Dornsife School of Public Health, Drexel University, Philadelphia, Pennsylvania, United States of America; 2 Northern Research Station, United States Department of Agriculture—Forest Service, Philadelphia, Pennsylvania, United States of America; 3 Department of Epidemiology and Biostatistics, Dornsife School of Public Health, Drexel University, Philadelphia, Pennsylvania, United States of America; 4 Department of Civil, Architectural, and Environmental Engineering, College of Engineering, Drexel University, Philadelphia, Pennsylvania, United States of America; 5 Division of Disease Control, Philadelphia Department of Public Health, Philadelphia, Pennsylvania, United States of America; Columbia University, UNITED STATES

## Abstract

Runoff from heavy precipitation events can lead to microbiological contamination of source waters for public drinking water supplies. Philadelphia is a city of interest for a study of waterborne acute gastrointestinal illness (AGI) because of frequent heavy precipitation, extensive impervious landcover, and combined sewer systems that lead to overflows. We conducted a time-series analysis of the association between heavy precipitation and AGI incidence in Philadelphia, served by drinking water from Delaware River and Schuylkill River source waters. AGI cases on each day during the study period (2015–2017) were captured through syndromic surveillance of patients’ chief complaint upon presentation at local emergency departments. Daily precipitation was represented by measurements at the Philadelphia International Airport and by modeled precipitation within the watershed boundaries, and we also evaluated stream flowrate as a proxy of precipitation. We estimated the association using distributed lag nonlinear models, assuming a quasi-Poisson distribution of the outcome variable and with adjustment for potential confounding by seasonal and long-term time trends, ambient temperature, day-of-week, and major holidays. We observed an association between heavy precipitation and AGI incidence in Philadelphia that was primarily limited to the spring season, with significant increases in AGI that peaked from 8 to 16 days following a heavy precipitation event. For example, the increase in AGI incidence related to airport precipitation above the 95^th^ percentile (vs no precipitation) during spring reached statistical significance on lag day 7, peaked on day 16 (102% increase, 95% confidence interval: 16%, 252%), and declined while remaining significantly elevated through day 28. Similar associations were observed in analyses of watershed-specific precipitation in relation to AGI cases within the populations served by drinking water from each river. Our results suggest that heavy precipitation events in Philadelphia result in detectable local increases in waterborne AGI.

## Introduction

The frequency of heavy precipitation events is increasing in many regions of the world as a consequence of climate change. The Northeastern United States has been particularly impacted relative to other parts of the country; the region saw a 58% increase in the number of days with heavy precipitation between 1958 and 2007 [1], and further increases of up to 22% have been projected to occur by the late 21^st^ century [2]. Heavy precipitation has the potential to introduce pathogens into water sources via increased runoff, and people may be exposed to these pathogens through direct contact with contaminated water or by drinking water. Heavy precipitation has been associated with increased risk of acute gastrointestinal illness (AGI) in epidemiologic studies conducted in developed countries such as the US, Canada, Taiwan, France, England, and Australia [3]. The association may suggest microbial contamination of drinking water supplies that evades elimination by water system treatments, making this an important public health research concern. While AGI increases in the epidemiologic studies have rarely been attributed to specific pathogens, candidates include microbes that are fairly resistant to drinking water disinfection, such as *Cryptosporidium* spp.

Some cities are more vulnerable than others to health-related concerns from heavy precipitation events, depending on source waters for drinking water supplies, upstream land use, impervious landcover, and the presence of combined sewer systems. A study performed in Massachusetts reported a positive association between extreme rainfall and emergency department visits for AGI, only in regions where combined sewer overflows (CSOs) impacted drinking water sources [4]. Philadelphia is a city of interest for the impact of heavy precipitation, due to increasing frequency of such events, use of surface water sources for the drinking water supply, a relatively high amount of impervious landcover [5], and the presence of combined sewers systems. Two time-series studies conducted in Philadelphia in the late 1990s found an association between turbidity of the local public water supply (as a proxy for microbial contamination) and AGI incidence in children and the elderly [6–7]. The levels of turbidity in Philadelphia drinking water have been reduced since the previous time-series studies were conducted through improvements in the water filtration system [8], but there has been no follow-up epidemiologic research of AGI in relation to turbidity or with precipitation events that may affect turbidity.

We studied the association between heavy precipitation events and AGI incidence in Philadelphia. We were interested in the predictive value of heavy precipitation events, as well as the streamflow of local rivers as an indicator of stormwater runoff. Evaluation of public health consequences from heavy precipitation is important in the face of observed and forecasted increases in the Northeastern US and the expected impact on urban area water supply systems [1].

## Materials and methods

**Fig 1:**
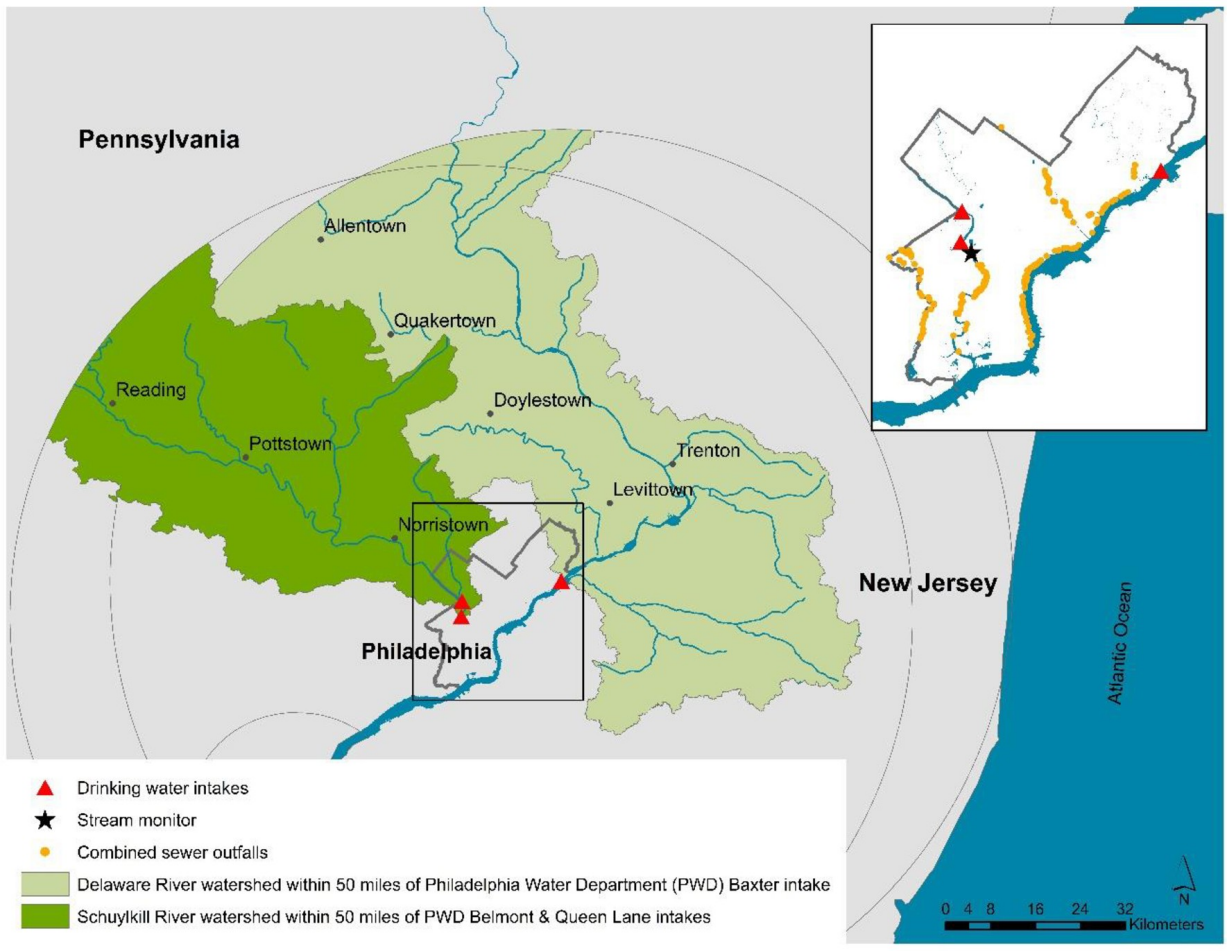
City of Philadelphia study region showing the Schuylkill River and Delaware River watershed areas upstream of Philadelphia Water Department intakes.

We conducted a time-series analysis of the association between daily precipitation and daily counts of AGI in the city of Philadelphia, over a three-year study period from January 1, 2015 through December 31, 2017. Our hypothesis was that heavy precipitation is associated with increases in AGI in the days following a precipitation event. The City of Philadelphia takes drinking water from two sources–the Schuylkill River and the Delaware River (Fig 1). These waters are treated in three plants operated by the Philadelphia Water Department (PWD) prior to distribution as the public water supply. Schuylkill River water is taken in at two locations and is treated at the Queen Lane plant, primarily serving Northwest Philadelphia, or the Belmont plant, primarily serving West Philadelphia. Delaware River water is treated at the Baxter plant, which primarily serves Northeast Philadelphia, Center City, and South Philadelphia. Certain areas of the city receive predominantly mixed water supplies. We cross-referenced a PWD service area map [8] with a map of Philadelphia zip codes to classify city zip codes according to primary drinking water source as Schuylkill River (18 total), Delaware River (17 total), and mixed supply (12 total).

AGI cases were identified using a syndromic surveillance system from the Philadelphia Department of Public Health (PDPH), in which the chief complaint at emergency department (ED) visits was used to classify cases as characteristic of ‘diarrhea’ and/or ‘vomiting’ syndromes. This research was determined to be exempt from review by the PDPH Institutional Review Board (IRB), the IRB of record for this study. We combined cases of diarrhea and vomiting syndromes on each day to tabulate an overall daily AGI count; if one ED visit was classified with both diarrhea and vomiting syndromes, the visit was counted as only one case of overall AGI. The PDPH syndromic surveillance data also included information on patient age group (0–17 years, 18–64 years, ≥65 years) and zip code of residence. We examined the distribution of daily total ED visit counts reported to the syndromic surveillance system (inclusive of ED visits for any reason, not only AGI) in order to identify outliers that could indicate reporting issues. There were 5 low outliers, defined as lower than the 25^th^ percentile value by more than 3 times the interquartile range. We imputed diarrhea and vomiting syndrome counts for those dates according to a 15-day moving average of total ED visits multiplied by the proportion of total ED visits that were diarrhea or vomiting syndrome visits on those days.

We used several sources of environmental data in our study. Climatic data collected by the National Oceanic and Atmospheric Administration [9] were obtained for the monitoring station located at the Philadelphia International Airport (PHL), including daily summary values for precipitation, maximum temperature, and minimum temperature. From hourly measurement data, we also calculated maximum hourly precipitation within a day. We summarized daily precipitation falling within the watershed boundaries for Philadelphia source waters using gridded precipitation data available from the Unified Precipitation Project of the NOAA Climate Prediction Center [10]. These data are created by smoothing data from multiple monitors, and are summarized within 0.25-degree latitude x 0.25-degree longitude grids. We delineated watershed boundaries for the Delaware River and Schuylkill River drainage basins using digital elevation models obtained from the US Geological Survey, for each river upstream from each PWD intake (Fig 1; the Schuylkill River watershed delineated from the either Queen Lane or Belmont treatment plant intakes was virtually identical, due to the proximity of the intakes and the scale of the data). Within each watershed, we summarized the gridded precipitation data as the daily mean and the daily hourly maximum–both for the total watershed area upstream from the PWD intake, as well as for the watershed area within a 50-mile (80.5 km) buffer upstream from the intake (a distance arbitrarily selected to define a proximal region within the larger watershed that would be more likely to impact local conditions in Philadelphia). Spatial analyses were conducted using ArcGIS software (v10.5; Redlands, CA).

We used streamflow (i.e., river flowrate in m^3^/s) as an indicator of stormwater runoff. Streamflow data were available from monitoring conducted at sites on the Schuylkill River (USGS 01474500 Schuylkill River at Philadelphia, PA) and the Delaware River (USGS 01463500 Delaware River at Trenton, NJ). We obtained these data online through the USGS National Water Information System [11] and by personal request to the USGS and PWD. The Schuylkill River monitoring site was located close to the PWD treatment plant intakes for Queen Lane (4.9 km distance) and Belmont (2.5 km distance, Fig 1). The Trenton monitoring site, located approximately 27.5 km upstream from the PWD Baxter intake, was selected to reflect Delaware River source water conditions, as it was the closest site with complete data during the study period located upstream of tidal conditions impacting monitoring sites closer to the intake (tidal conditions would be expected to influence streamflow in ways that are not directly related to stormwater).

A ‘heavy precipitation event’ was defined as daily rainfall greater than the 95th percentile of rainfall on all days during the study period. Daily ‘precipitation’ variables considered were total precipitation at the Philadelphia International Airport (PHL), maximum hourly precipitation at PHL, average precipitation within the watershed, and maximum hourly precipitation within the watershed, as well as mean streamflow as an indicator of stormwater runoff.

To estimate the association between precipitation and AGI, we fit distributed lag generalized linear models (GLM) in R (version 3.5.1), using the dlnm package (version 2.3.5). We fit the models assuming an overdispersed (quasi) Poisson distribution with a log link, in which the outcome–daily count of AGI–was regressed on precipitation, with adjustment for confounding by daily temperature, day-of-week, holidays, seasonal patterns, and long-term time trends. Lagged associations between the outcome at time *t* and measurements of precipitation and temperature at times *s<t* were considered with a distributed lag model [12]; a maximum lag of 28 days was defined *a priori*, based on the literature [13–14].

We first developed a base model including only the covariates as predictors of AGI; we decided on the functional forms for the nonlinear associations between predictors and AGI and for the lag functions within the distributed lag model, along with their associated degrees of freedom, using a combination of quasi AIC (QAIC) [12, 15] and analysis of residual plots to identify residual seasonal or long-term temporal variation (residual plot for base model is shown as S1 Fig). Average daily temperature was modeled as a natural cubic spline with 3 degrees of freedom. Day-of-week (Mon, Tues, Weds, etc.) and holidays (yes/no) were modeled as indicator variables. Long-term time trends were modeled using a variable with values for sequential day of the study period, using a natural spline model with 21 degrees of freedom (7 df/year, as in [16]). Seasonal patterns were modeled using a variable for day of the calendar year, using a natural spline model with 7 degrees of freedom. A separate indicator variable for season was also included because this resulted in a lower value for the QAIC (season defined by calendar month; winter: Dec-Feb, spring: Mar-May, etc.).

Each precipitation variable was added to the base model, separately, for estimation of its association with AGI. We compared model fit with different precipitation variables by the QAIC. Each precipitation variable was categorized and modeled with an indicator variable to estimate a relative rate (RR) and 95% confidence interval (CI) for AGI incidence in association with daily precipitation values higher than the 95^th^ percentile versus incidence at the median precipitation level or below. The models also included a separate indicator variable for a mid-level amount of precipitation, from greater than the median to the 95^th^ percentile. In secondary analyses, we modeled the flexible, non-linear association between precipitation and AGI, with precipitation modeled as a natural cubic spline with 4 degrees of freedom. In addition to confounders included in the base model (temperature, day-of-week, holiday, seasonal patterns, long-term time trends), we evaluated confounding of daily precipitation variables with the cumulative precipitation in the past 4 weeks (based on measurement at PHL), with the assumption that soil moisture conditions preceding a heavy precipitation event would affect permeability and, therefore, runoff, and furthermore, that a ‘first flush’ rainfall following a dry period would contain more contaminants and pathogens than heavy rainfall following a wet period. As a sensitivity analysis, we analyzed the impact of using alternative cutpoints–the 90^th^ and 99^th^ percentiles–as cutoffs for defining a heavy precipitation event. Finally, we checked the sensitivity of results to alternative degrees of freedom for seasonal patterns (day-of-calendar year) and temperature, by doubling and halving (rounded up) the degrees of freedom from our base model.

Separate models were constructed for populations in three study regions: 1) Philadelphia overall; 2) zip codes with Delaware River source water; and, 3) zip codes with Schuylkill River source water. Zip codes with predominantly mixed supplies were excluded from analyses by source water. In models for the two source water areas, we considered the corresponding river-specific exposure variables of daily within-watershed precipitation and streamflow. In models for Philadelphia overall, we considered daily airport precipitation, combined watershed precipitation calculated as an average of daily precipitation within each of the two watersheds, and combined streamflow calculated as the average streamflow of the two rivers. Models for each region were also fitted separately by season (because exposures may differ by season) and age group (because the very young and very old have higher rates of AGI than other age groups and may differ in susceptibility to AGI from heavy precipitation). We also evaluated diarrhea and vomiting cases of AGI as separate outcomes in the main analysis (Philadelphia overall, year-round) and in subanalyses in which we found a statistically significant increase for all AGI.

## Results

**Fig 2:**
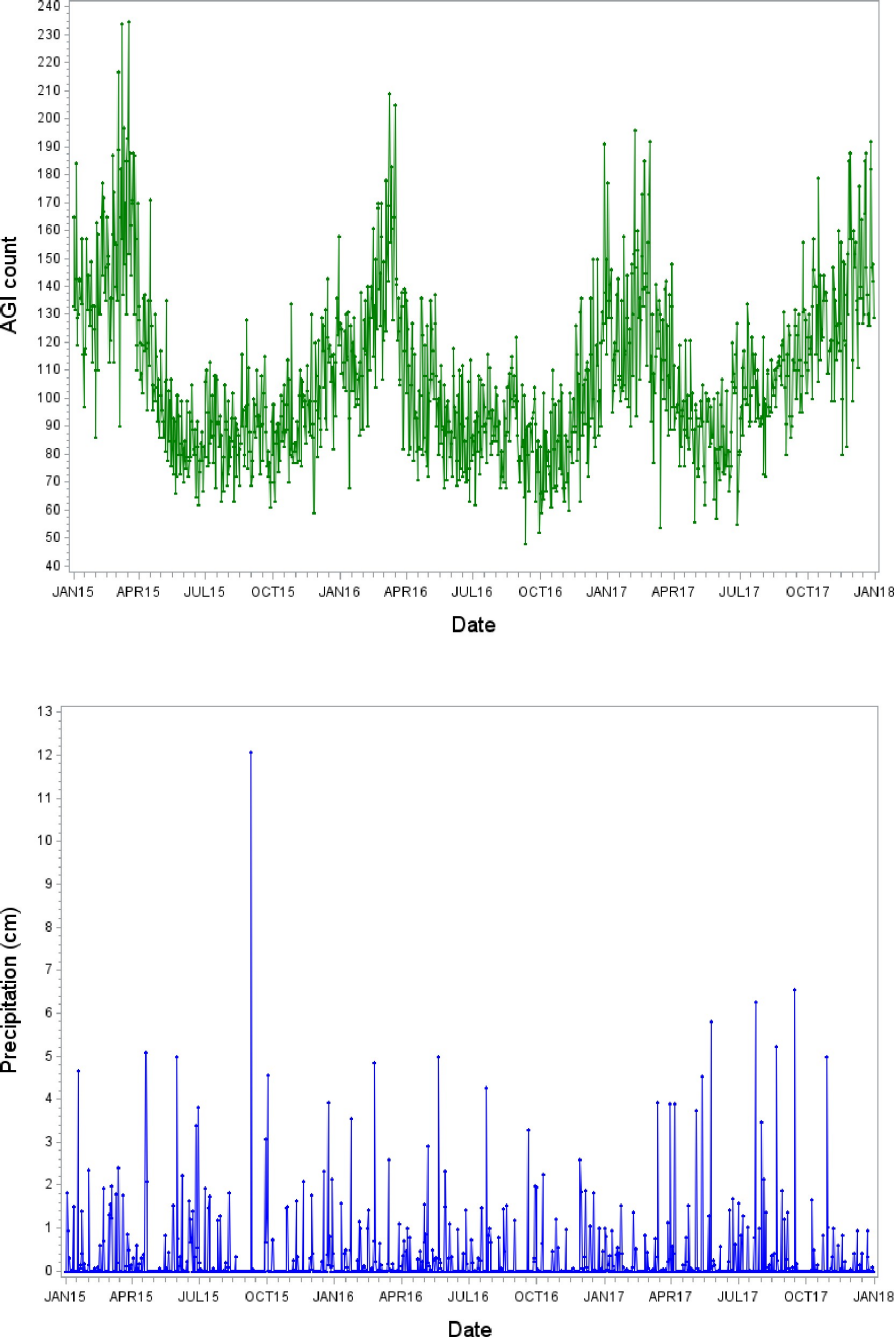
Timeseries plots of daily values during the study period, for: a) AGI counts identified by syndromic surveillance; b) total precipitation recorded at the Philadelphia International Airport.

The daily count of AGI cases during the study period ranged from 48 to 235, citywide, with a median of 102 (Table 1). On a typical day (the median of each distribution), there were more vomiting cases (88) than diarrhea cases (25). Daily AGI counts were typically highest in winter (128) and lowest in summer (91), with a clear annual pattern (Fig 2A), although the highest count during the study period (235) occurred in spring (March) of 2015. Daily counts were typically highest among persons ages 18 to 64 and persons living in the PWD Baxter plant service area–both predominantly reflective of underlying population counts in these subgroups. Precipitation fell on fewer than 32% of the days in the study period, as measured by the monitor at PHL. Precipitation amounts were 1.68 cm at the 95^th^ percentile, 4.67 cm at the 99^th^ percentile, and a maximum of 12.1 cm, which occurred in September of 2015 (Fig 2B). On days with any rainfall recorded at PHL, 50% had totals of 0.406 cm or less. The highest values of daily mean precipitation within the 50-mile watersheds were lower than measured at PHL, presumably due to averaging and smoothing of monitor-based data over a large area, with maximums of 7.35 cm in the Delaware River watershed and 6.04 cm in the Schuylkill River watershed (vs 12.1 cm at PHL). As expected, streamflow of the Delaware River was greater than that of the Schuylkill River (195 m^3^/s vs. 47.8 m^3^/s at the median of daily values).

**Table 1:** Daily distributions of AGI visits and environmental variables during the study period, 2015–2017.

Percentile	0p	25p	50p	75p	90p	95p	100p
AGI visits (daily count)							
All AGI	48	87	102	126	145	163	235
Diarrhea cases	8	20	25	32	40	47	93
Vomiting cases	42	74	88	108	128	142	200
By season							
Winter	68	110	128	144	161	174	196
Spring	54	89	102	129	157	182	235
Summer	55	80	91	104	111	116	134
Fall	48	82	95	114	134	146	188
By age							
<18 years	3	25	35	50	64	73	113
18–64 years	21	49	57	66	75	81	108
≥65 years	0	8	10	13	15	17	24
By primary drinking water source							
Delaware River	16	39	47	58	71	79	120
Schuylkill River	9	30	36	43	51	58	82
Environmental variables (daily value)							
Temperature, minimum (˚C)	-16.7	2.2	10.0	18.9	22.2	23.3	27.8
Temperature, maximum (˚C)	-8.3	11.1	20.0	28.3	31.7	33.3	36.7
Precipitation, total at PHL (cm)	0	0	0	0.076	0.940	1.68	12.1
Precipitation on rainfall days, total at PHL (cm)	0.025	0.102	0.406	1.22	2.13	3.89	12.1
Precipitation, 1-hour maximum at PHL (cm)	0	0	0	0.051	0.305	0.610	3.708
Precipitation, mean within 50-mile Delaware River watershed (cm)	0	0	0.013	0.214	1.05	1.69	7.35
Precipitation, mean within 50-mile Schuylkill River watershed (cm)	0	0	0.001	0.177	1.06	1.87	6.04
Streamflow, Delaware River (m^3^/s)	67.1	123	195	303	470	600	1877
Streamflow, Schuylkill River (m^3^/s)	9.66	30.4	47.8	74.0	128	178	827

AGI = acute gastrointestinal illness; p = percentile

Table 2 shows a summary of associations between environmental variables and AGI incidence in Philadelphia. Each RR compares values >95^th^ percentile to those at the median and below (the medians were 0 cm for PHL total precipitation and 0.186 cm for precipitation in the 50-mile combined watershed), and we note the first peak of an increased rate across lag days (i.e., the highest RR in a trend of increasing risk across lags; we specify the first peak because some associations peaked a second time toward the end of the 28-day lag period). Heavy precipitation was weakly associated with AGI in Philadelphia, for both total precipitation measured at PHL (peaking at 1.4% increase on lag day 10, 95% CI: -0.9% to 3.8%) and mean precipitation in the combined 50-mile watershed (peaking at 2.0% increase at lag 17, 95% CI: -0.5% to 4.6%). Heavy precipitation was associated with increased incidence of diarrhea AGI, specifically. For example, diarrhea incidence was increased by 4.3% (95% CI: 0.4% to 8.2%) with PHL precipitation above the 95^th^ percentile at lag day 9; the effect size was similar for the combined 50-mile watershed (4.9% increase), but the peak occurred with a longer lag of 18 days. Estimated RRs were higher for children (age <18 years) than for other age groups, but without statistical significance for the PHL and watershed precipitation variables. The result for combined watershed precipitation and all AGI (2% increase at lag 17) was robust to the 99^th^ percentile as an alternate cutpoint (2.3% increase at lag 17) and was diminished when setting the cutpoint as the 90^th^ percentile (0.4% increase at lag 7) (S1 Table). With adjustment for 4-week cumulative precipitation (S2 Table), the associations with all AGI, diarrhea, and vomiting were slightly strengthened and the association of combined watershed precipitation with all AGI attained statistical significance (RR = 1.040, 95% CI: 1.002–1.080). However, there was no indication by comparing QAIC values that inclusion of cumulative precipitation improved the fit of the models.

**Table 2:** Association of daily precipitation and streamflow variables with AGI incidence, comparing AGI at exposure values >95^th^ percentile to ≤median (first RR peak after lag 0 shown).

	N events	Exposure–Precipitation at Philadelphia International Airport		Exposure–Precipitation within combined 50-mile watershed^a^		Exposure–Combined streamflow^b^	
Model^c^		Lag with first peak (days)	RR (95% CI)	Lag with first peak (days)	RR (95% CI)	Lag with first peak (days)	RR (95% CI)
All AGI	117,937	Lag 10	1.014 (0.991, 1.038)	Lag 17	1.020 (0.995, 1.046)	Lag 9	1.015 (1.004, 1.027)
Diarrhea cases	29,641	Lag 9	1.043 (1.004, 1.082)	Lag 18	1.049 (1.008, 1.092)	Lag 9	1.017 (0.999, 1.035)
Vomiting cases	101,738	Lag 11	1.006 (0.983, 1.030)	Lag 14	1.011 (0.987, 1.035)	Lag 10	1.018 (1.007, 1.030)
By age							
<18 years	42,494	Lag 12	1.020 (0.978, 1.065)	Lag 19	1.049 (0.997, 1.103)	Lag 9	1.037 (1.016, 1.059)
18–64 years	63,665	Lag 10	1.016 (0.993, 1.039)	Lag 10	1.015 (0.991, 1.039)	Lag 9	1.002 (0.990, 1.013)
≥65 years	11,588	NA*		Lag 18	1.028 (0.975, 1.083)	NA	
By season							
Winter	34,675	Lag 8	1.177 (0.866, 1.601)	Lag 9	1.176 (0.849, 1.630)	Lag 12	1.101 (0.824, 1.470)
Spring	30,828	Lag 16	2.024 (1.163, 3.523)	Lag 11	5.839 (2.600, 13.11)	Lag 8	1.402 (0.924, 2.127)
Spring—Diarrhea	8204	Lag 15	3.774 (1.590, 8.960)	Lag 11	8.441 (2.308, 30.86)	Lag 9	1.833 (0.907, 3.701)
Spring—Vomiting	26,709	Lag 16	1.824 (1.030, 3.228)	Lag 11	5.962 (2.601, 13.67)	Lag 8	1.391 (0.908, 2.130)
Summer	25,264	NA	No peak	NA	RRs<1^d^	NA	No peak
Fall	27,170	NA	No peak	NA	No peak	NA	No observations >95p

AGI = acute gastrointestinal illness; RR = relative rate; CI = confidence interval; NA = not applicable due to no peak, RRs<1, or model did not converge

^a^Average of daily mean precipitation within Delaware and Schuylkill River watersheds

^b^Average of Delaware River and Schuylkill River daily mean streamflow values

^c^All estimates are adjusted for temperature, day-of-week, holidays, and temporal trends (natural spline variables for consecutive day of the study and day of the calendar year). Non-season specific estimates are also adjusted for season using indicator variables.

^d^Inverse association disappeared with adjustment for 4-week cumulative precipitation

**Fig 3:**
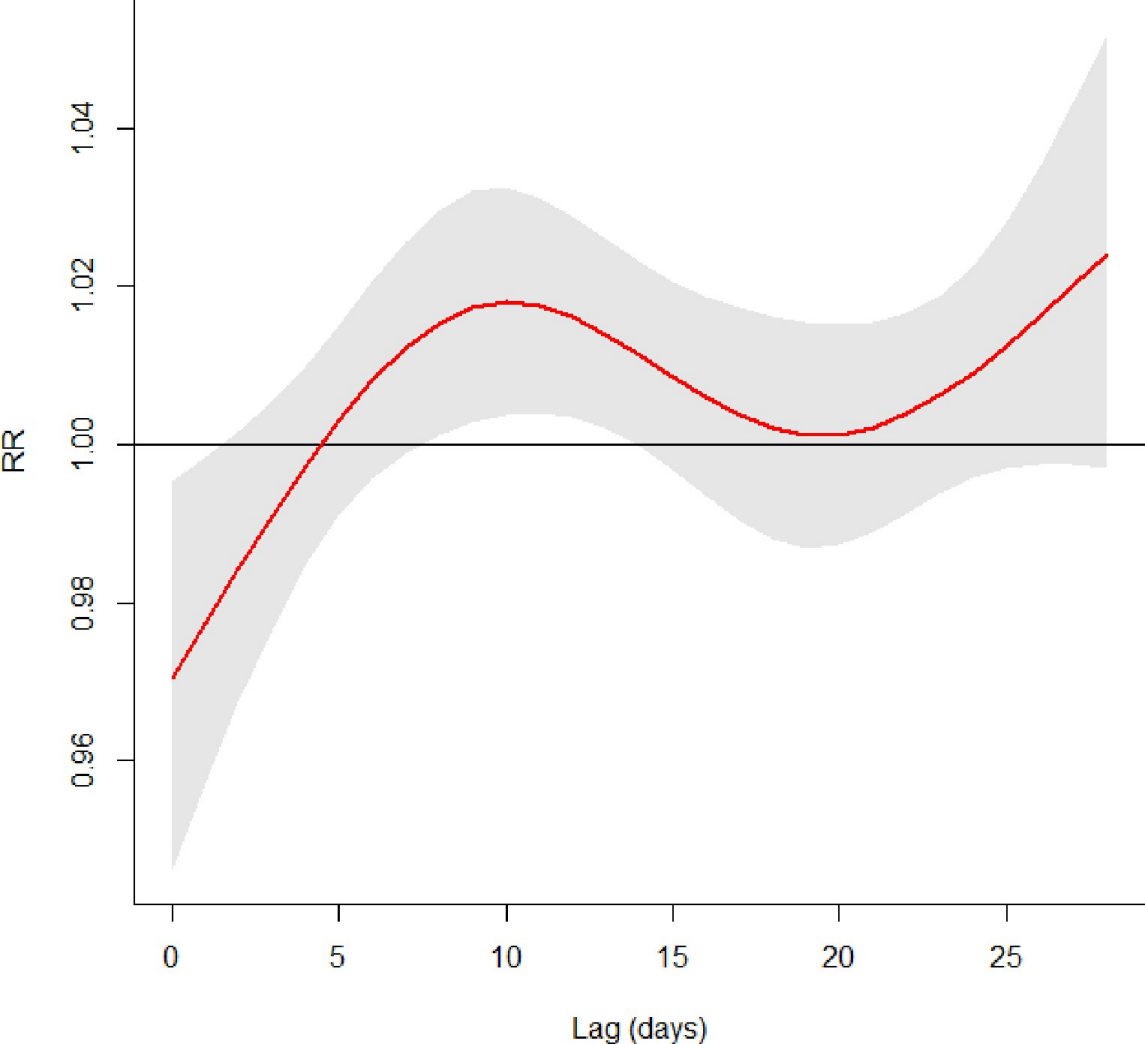
Association between daily combined streamflow (average of Schuylkill and Delaware River streamflow values) and AGI incidence in Philadelphia across lag days, comparing AGI at streamflow levels >95th percentile to ≤median (RRs and 95% confidence interval shown), with adjustment for temperature, day-of-week, holidays, season, and temporal trends (natural spline variables for consecutive day of the study and day of the calendar year).

High combined streamflow (average of Delaware and Schuylkill River mean flow rates) was associated with significantly increased incidence of all AGI in Philadelphia on days 7 through 13 following streamflow above the 95^th^ percentile, compared with streamflow at the median or below (Fig 3); the association peaked on lag day 9 (Table 2), with an estimated 1.5% increase in AGI (95% CI: 0.4%, 2.7%). The effect size for combined streamflow was similar for diarrhea and vomiting cases. The observed association was similar when high streamflow was defined as values above the 90^th^ percentile (peak increase of 1.6% on lag day 9), and was stronger when using the 99^th^ percentile as the cutpoint (peak increase of 2.4% on lag day 17). Combined streamflow was significantly associated with AGI incidence in children, with a 3.7% increase in AGI that peaked 9 days after streamflow above the 95^th^ percentile. There was little change in the relationship between combined streamflow and all AGI, diarrhea, or vomiting with adjustment for 4-week cumulative precipitation (S2 Table).

**Fig 4:**
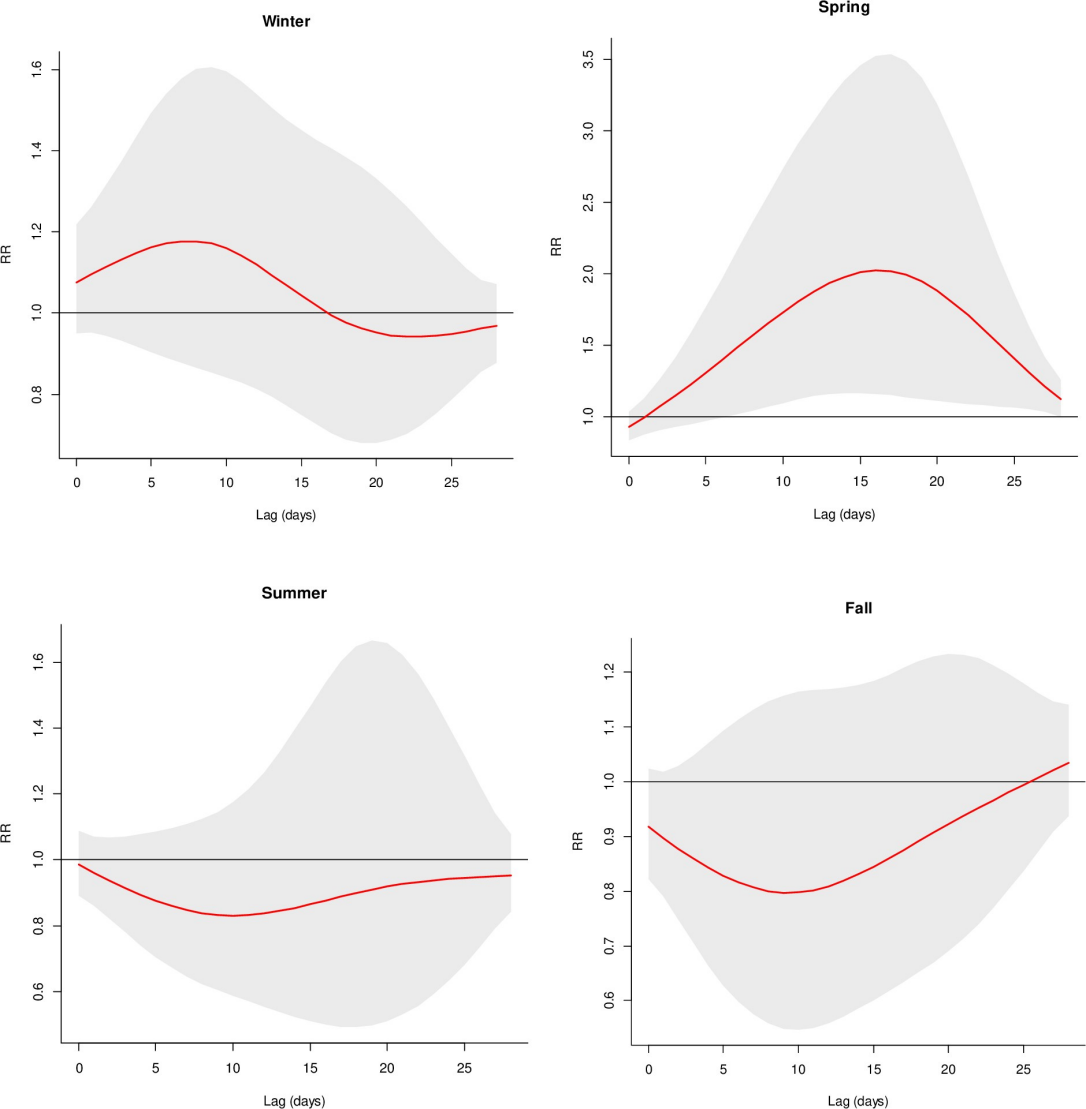
Association of daily precipitation (PHL) with AGI incidence, comparing AGI at exposure values >95^th^ percentile to ≤median (first RR peak after lag 0 shown), across lags and by season (smoothed RRs in red, smoothed 95% CIs in grey) with adjustment for temperature, day-of-week, holidays, and temporal trends (natural spline variables for consecutive day of the study and day of the calendar year).

Increases in AGI with heavy precipitation and high streamflow were most notable in the spring season (Table 2 and Fig 4). For example, total precipitation (PHL) above the 95^th^ percentile during spring was associated with peak increases of 102% for all AGI (95% CI: 16%, 252%), 277% for diarrhea (95% CI: 59%, 796%), and 82% for vomiting (95% CI: 3%, 222%), relative to AGI incidence at the median precipitation value of 0 cm. AGI increases related to heavy precipitation (PHL) in spring reached statistical significance on lag day 7, peaked on day 16, and declined while remaining significantly elevated through day 28 (the end of our lag period, Fig 4). Weaker, non-significant increases were observed during the winter months, and there was no association in summer or fall (Fig 4). Associations with precipitation in the combined 50-mile watershed were notably elevated in spring, but the estimates were imprecise (Table 2), such as a 484% increase in AGI on lag day 11 (95% CI: 160%, 1211%). The association with combined watershed precipitation was stronger with levels above the 99^th^ percentile (658% increase) and lower with levels above the 90^th^ percentile (63% increase)–both with statistical significance (S1 Table). The association with streamflow was also strongest in spring (40% increase on lag day 8, Table 2), but this increase was not statistically significant. Season-specific models in spring and summer were importantly affected by adjustment for cumulative 4-week precipitation (S2 Table). With the adjustment, associations with combined watershed precipitation remained strongly and significantly elevated in spring, although the estimate for diarrhea was very imprecise, with high upper confidence limits. An observed, statistically significant inverse association between combined watershed precipitation and AGI during summer was changed to a notably null association with the adjustment for cumulative precipitation, and the summer association between PHL precipitation and AGI changed from a notably null association with no peak to a small, non-significant increase with the adjustment. Lower QAICs for models including the cumulative precipitation covariate in spring and summer also indicated the importance of this adjustment. Estimates for heavy precipitation in winter and fall were not strongly confounded by cumulative precipitation. The modest, non-significant increase seen with streamflow in spring (RR = 1.4) was diminished to no association upon adjustment for cumulative precipitation (S2 Table).

In analyses limited to the study regions (zip codes) served by source water from each river, the highest RRs were again seen in the spring. These increases were statistically significant for precipitation within each watershed (Table 3), but not for river-specific streamflow. For example, for the region with Delaware River source water, there was a peak estimated 210% increase in AGI (95% CI: 21%, 699%) on lag day 11 associated with daily precipitation above the 95^th^ percentile (vs below the median) during the spring season, for mean precipitation in the 50-mile watershed upstream of the PWD Baxter intake. The corresponding analysis for the region with Schuylkill River source water found that mean precipitation in the 50-mile watershed upstream of the PWD Queen Lane and Belmont intakes was associated with an estimated 172% increase in AGI (95% CI: 27%, 481%), with 12 days lag. As seen in Philadelphia, overall, the association seen in the spring was inversed in the summer months, with reductions in AGI incidence associated with heavy precipitation, and these inverses disappeared with adjustment for cumulative precipitation.

**Table 3:** Association of daily mean precipitation within each 50-mile watershed with AGI incidence by primary drinking water source, comparing AGI at precipitation levels >95^th^ percentile to ≤median (first RR peak after lag 0 shown).

Model^a^	N events	Lag with first peak (days)	RR (95% CI)
Delaware River			
All AGI	54,551	28	1.025 (0.987, 1.065)
Diarrhea	13,788	8	1.016 (0.965, 1.069)
Vomiting	47,231	28	1.021 (0.982, 1.062)
By season			
Winter	16,247	4	1.041 (0.757, 1.433)
Spring	14,079	11	3.104 (1.205, 7.996)
Spring, Diarrhea	3805	13	34.28 (6.142, 191.32)
Spring, Vomiting	12,223	10	1.954 (0.776, 4.919)
Summer	11,690	NA	No peak
Fall	12,535	NA	No peak
Schuylkill River			
All AGI	40,843	19	1.019 (0.994, 1.044)
Diarrhea	10,061	19	1.029 (0.983, 1.077)
Vomiting	35,333	19	1.015 (0.988, 1.042)
By season			
Winter	11,789	17	1.177 (0.767, 1.808)
Spring	10,942	12	2.719 (1.272, 5.811)
Spring, Diarrhea	2874	13	3.311 (0.780, 14.06)
Spring, Vomiting	9473	11	2.681 (1.235, 5.821)
Summer	8665	NA	RRs<1^b^
Fall	9447	NA	No peak

AGI = acute gastrointestinal illness; RR = relative rate; CI = confidence interval; NA = not applicable due to no peak or RRs<1

^a^All estimates are adjusted for temperature, day-of-week, holidays, and temporal trends (natural spline variables for consecutive day of the study and day of the calendar year). Non-season specific estimates are also adjusted for season using indicator variables.

^b^Inverse association disappeared with adjustment for 4-week cumulative precipitation

**Fig 5:**
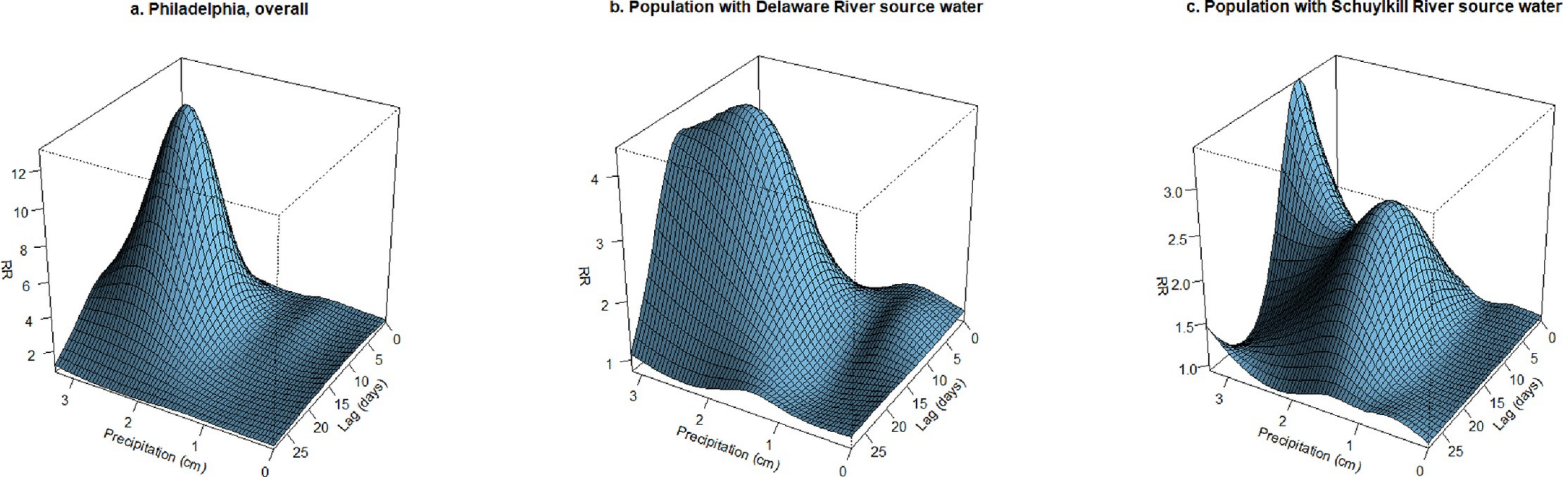
Association between daily precipitation and AGI incidence in Philadelphia during the spring season across lag days, fit using natural spline with comparison of AGI at each precipitation level to the median level (exposure estimated as mean precipitation in 50-mile watershed region), with adjustment for temperature, day-of-week, holidays, and temporal trends (natural spline variables for consecutive day of the study and day of the calendar year), for: a) Philadelphia, overall; b) Population with Delaware River source water; c) Population with Schuylkill River source water.

Models considering flexible relationships across the continuum of 50-mile watershed precipitation during the spring season are shown in Fig 5 for the comparison of AGI at each precipitation level to the median precipitation, across lags. These three-dimensional plots show increasing AGI incidence by increasing precipitation levels that rose in the days following a rainfall event, peaked with the highest RRs appearing on lag days 13 (Philadelphia, overall), 13 (Delaware) and 8 (Schuylkill), and declined with longer lags. For example, AGI incidence in the region with Delaware River source water peaked on lag day 13 at 2.5 cm precipitation (relative to AGI at the median precipitation level of 0.001 cm), with an increase of 342% (95% CI: 6.3%, 1739%). For the region with Schuylkill River source water, AGI incidence peaked on lag day 8 at the highest level of spring season precipitation in the watershed (3.3 cm), with an increase of 243% (95% CI: -7.3%, 432%) relative to AGI at the median precipitation level (0.013 cm). The patterns of association shown in Fig 5 were similar with adjustment for 4-week cumulative precipitation, and the peak association for the Schuylkill River watershed became statistically significant.

In additional analyses, variables for maximum hourly precipitation at PHL or in the watersheds were less, or similarly predictive of AGI incidence than mean measures. Associations with precipitation in the entire watershed (i.e., not limited to 50 miles upstream) were similar to those with the 50-mile metrics, both for the combined watershed and the Schuylkill River watershed. In analyses of the Delaware River watershed, models of precipitation in the entire watershed had lower QAIC values than with precipitation limited to the 50-mile buffer; however, the entire watershed exposure resulted in RRs that were statistically significant, but notably inflated and very imprecise. For this reason, we decided to present the more modest results for the 50-miile buffer precipitation. Estimated rate increases were robust to alternative degrees of freedom for seasonal patterns or temperature in analyses of watershed precipitation for Philadelphia overall (S1 Table) and the Schuylkill River Watershed (not shown). The patterns of association in the Delaware River Watershed were very similar with alternative degrees of freedom, although some results were no longer statistically significant when using 6 df for temperature or 4 df for season.

## Discussion

Our study brought together several sources of data to address our central hypothesis, that heavy precipitation is associated with increased incidence of AGI in Philadelphia, in the days following the precipitation event. In addition to the hypothesis we tested, we postulated that any associated increase occurred via waterborne transmission. We found an association between heavy precipitation and AGI incidence in Philadelphia that was primarily limited to the spring season. Heavy precipitation was consistently associated with AGI in spring, whereas AGI increases with spring streamflow were not statistically significant. In our consideration of up to 28 days lag, we found significant increases in AGI during spring that peaked from 8 to 16 days following a heavy precipitation event.

A mechanistic pathway involving waterborne transmission of AGI begins with heavy precipitation causing higher than normal concentrations of pathogens to enter source waters, either with stormwater runoff or from CSOs. Stream bottom resuspension of sediment is also likely to play a role [17]. Philadelphia residents may then be exposed through direct contact with river water/sewage or by ingestion of contaminated drinking water, although the plausibility and importance of these pathways have not been adequately investigated. Philadelphia’s combined sewer system covers almost two-thirds of the sewer service area and discharges an estimated 16 billion gallons of overflow effluent per year [18]; however, the city’s 164 combined sewer outfalls are located downstream of river water intakes for the drinking water supply [19] (Fig 1). Nevertheless, Philadelphia CSOs remain a concern for the possibility of direct contact with contaminants, and CSOs in upstream municipalities are a relevant concern for contamination of Philadelphia’s drinking water sources.

As an example, we consider the plausibility of exposure pathways for *Cryptosporidium*, a frequent cause of waterborne gastroenteritis. Cryptosporidiosis has been associated with heavy precipitation in multiple studies, presumably due to contamination of water supplies with runoff and flooding [3, 13]. In an investigation of Delaware River water at Trenton, NJ, *Cryptosporidium* concentration was increased following precipitation [17], and was even more strongly correlated with other variables such as streamflow and turbidity.

Recreation in untreated rivers and lakes is a known cause of disease outbreaks [20], and studies of sporadic cryptosporidiosis have also identified freshwater swimming as a risk factor [21–22]. Recreational contact with local waters may have contributed to the increases in AGI we observed following heavy precipitation; however, the association was present only during the spring season when recreational contact is likely limited due to cold river water temperatures. Furthermore, we there was no association with heavy precipitation during the summer, when recreational contact is expected to be high. An additional exposure pathway involving direct contact with sewage may occur with heavy precipitation causing sewer lines to back-up into basements, but again, there is no reason that this scenario would be more likely to occur with heavy precipitation during spring. It is possible that seasonal differences in the relationship between heavy precipitation and AGI could result, not from differences in contact with water, but from differences in the presence of pathogens between seasons. For example, *Cryptosporidium* concentration was highest during winter and spring in the Delaware River survey at Trenton, NJ [17], coinciding with the highest river flow and turbidity, and consistent with the association we observed in the spring season.

Waterborne illness via ingestion of contaminated drinking water could occur if pathogen input from source waters evades treatment. *Cryptosporidium* is commonly detected at low levels in source waters for Philadelphia’s water supply [8], and was also frequently detected in a survey of filtered drinking water supplies [23]. *Cryptosporidium* can be resistant to drinking water disinfection with chlorine [24–25], particularly during its spore phase in which oocysts are encased by a thick wall. Outbreaks of cryptosporidiosis through drinking water have occurred primarily with suboptimal treatment; however, rates of sporadic cryptosporidiosis with treated water supplies are unknown [24]. Epidemiologic studies of the relationship between turbidity of finished water from public systems and AGI incidence rather consistently find a positive relationship [14], which may indicate risks due to residual microbial contamination of treated drinking water. Another pathway to contamination of drinking water by pathogens is within the distribution system, through cross-contamination of water distribution pipes by sanitary sewer pipes, which can occur under conditions of low water pressure or when there is a water main break; however, it is not clear that cross-contamination would be more likely to occur following heavy precipitation.

The highest AGI increases and most consistent results in our study were found with the exposure metric of precipitation falling within the local watersheds. These results were robust to all sensitivity analyses and adjustments, and demonstrated exposure-response relationships. Precipitation measured at the Philadelphia airport (PHL) was also associated with increased AGI incidence, but with less consistency across analyses. AGI increases observed with streamflow were not consistently elevated during the spring season, and any observed increases in spring were diminished with adjustment for 4-week cumulative precipitation. Interpretation of the differences between exposures is speculative. We created the watershed precipitation exposure metric to represent precipitation that would directly impact the Delaware- and Schuylkill River source waters for Philadelphia’s drinking water supply, via runoff into the rivers. In contrast, precipitation at PHL is measured at one watershed location below the PWD treatment plant intakes. The stronger and more consistent findings with watershed precipitation may indicate a relevant exposure pathway through contamination of source waters, and subsequently, the water supply. However, inconsistent associations with streamflow during spring suggest that runoff causing high streamflow is not an important part of the pathway. These findings may indicate that the input of pathogens to source waters is more important than the amount of runoff, itself. Observed AGI increases with precipitation and not streamflow might also indicate importance of an exposure pathway involving direct contact with contaminated stormwater in the city, such as through backed-up sewers, that can occur independently of streamflow levels. Further characterization of exposure pathways is needed to more fully understand the utility of particular exposure metrics for predicting AGI risks with heavy precipitation.

AGI increases associated with precipitation and streamflow variables in our study peaked at different lags following a heavy precipitation event, from 8 to 16 days. These lags represent the amount of time from the heavy precipitation event until presentation of a patient with AGI to the emergency department. For an exposure pathway involving drinking water, the lag time incorporates the time from river contamination until the drinking water reaches the consumer’s tap, incubation periods for common microbiological infections, and any delay in seeking medical care for AGI. Given the ecological nature of the time-series study design, the lag time represents the average lag within the area studied. We suspect that a lag time based on direct contact with contaminated stormwater would be shorter than for a pathway involving ingestion of contaminated drinking water, because of the timing of exposure after a heavy precipitation event. However, it is difficult to infer particular mechanisms based on lag times seen in our results, given disparate lags between exposure variables and between the entire year and the spring season. We are nevertheless assured that the peaks we observed are fairly consistent with those reported in similar research [3,14].

The AGI metric in our study, based on emergency department visits, is subject to lack of sensitivity, as typically only severe AGI cases will seek emergency care. Undercounting is common in timeseries studies that use patient visit counts as the dependent variable; this undercounting reduces the study power but will not cause bias as long as the variation in identified AGI cases is representative of the variation of the underlying true daily counts. It is also likely that persons with lower socioeconomic status use emergency care more frequently than the rest of the population (e.g., persons who do not have health insurance), and therefore these population groups are overrepresented in the AGI counts. This overcounting of a subpopulation would not affect our results unless there was a systematic difference in the association between precipitation and AGI among this subgroup (e.g., if this subgroup was more susceptible to waterborne AGI), compared to the rest of the population–and even then, it would not necessarily dismiss the overall association.

The syndromic surveillance system we used to identify AGI is designed for rapid identification of spikes in illness; this system has not been checked for accuracy of disease classification. Furthermore, the grouping of diarrhea and vomiting syndromes lacks detailed etiology. Nevertheless, syndromic surveillance data for gastroenteritis had a strong relationship with norovirus outbreaks in a US national database [26], indicating its utility for tracking major infectious illnesses. Furthermore, syndromic surveillance data has been used in other disease research; a study conducted in New York City reported that daily counts of the diarrhea syndrome (identified using the same algorithm as in Philadelphia) were associated with daily average turbidity of the drinking water supply [27]; interesting with comparison to our results, the association was only present during the spring months. There is a well-known peak of highly communicable viral agents in late winter/early spring, such as norovirus and rotavirus [26], and this clear seasonal pattern was apparent in the syndromic surveillance data we utilized in our study (Fig 2A). Cryptosporidiosis, in contrast, occurs predominantly in summer [28]. In our time-series analysis, we adjusted for predominant seasonal patterns of AGI within the calendar year, as well as adjusting for season (4 discrete seasons, as defined in our study). These adjustments appeared to effectively control for seasonal confounding of the relationship between heavy precipitation and AGI, as judged by a plot of residuals across the days of the study period (S1 Fig).

Our findings follow research conducted in Philadelphia by Schwartz et al. in the 1990s [6–7], in which turbidity of finished drinking water was associated with increased incidence of AGI in children and the elderly. We did not study turbidity directly, as we were not granted access to these data by the PWD (personal communication, PWD). Instead, we studied heavy precipitation as part of the same hypothesized mechanistic pathway as turbidity, although turbidity is a surrogate for microbial contamination whereas precipitation is a cause of microbial contamination. Our results suggest that despite improvements to Philadelphia’s drinking water supply since the Schwartz studies were conducted, such as notable reductions in turbidity since the 1990s [8], there may still be conditions under which detectable increases in AGI are caused via waterborne transmission.

We found an association between heavy precipitation events and AGI incidence in Philadelphia, limited to the spring season. While it is not possible to confirm the exposure pathway or implicated pathogens from our data, these details could be addressed in future research. Targeted sampling strategies to identify specific pathogens following heavy precipitation events during the spring season may provide evidence designating water as the medium of exposure and could help clarify the mechanistic pathway (e.g., source water, finished water, CSOs). Development of mitigation strategies will only be possible with further information.

## Supporting information

S1 Fig Residuals by day from base model. (DOCX)

S1 Table Association of daily precipitation and streamflow variables with AGI incidence, comparing AGI at exposure values >95^th^ percentile to ≤median (first RR peak after lag 0 shown), in crude model, primary model, and sensitivity analyses. (DOCX)

S2 Table Association of daily precipitation and streamflow variables with AGI incidence, comparing AGI at exposure values >95^th^ percentile to ≤median (first RR peak after lag 0 shown), in models with- and without adjustment for 4-week cumulative precipitation. (DOCX)
