# Supplementary figures and images for: Heavy precipitation, drinking water source, and acute gastrointestinal illness in Philadelphia, 2015-2017
Source: PLoS One. 2020 Feb 24;15(2):e0229258. doi: 10.1371/journal.pone.0229258 (PMC7039462; doi:10.1371/journal.pone.0229258)

**S1 Fig. Residuals by day from base model**


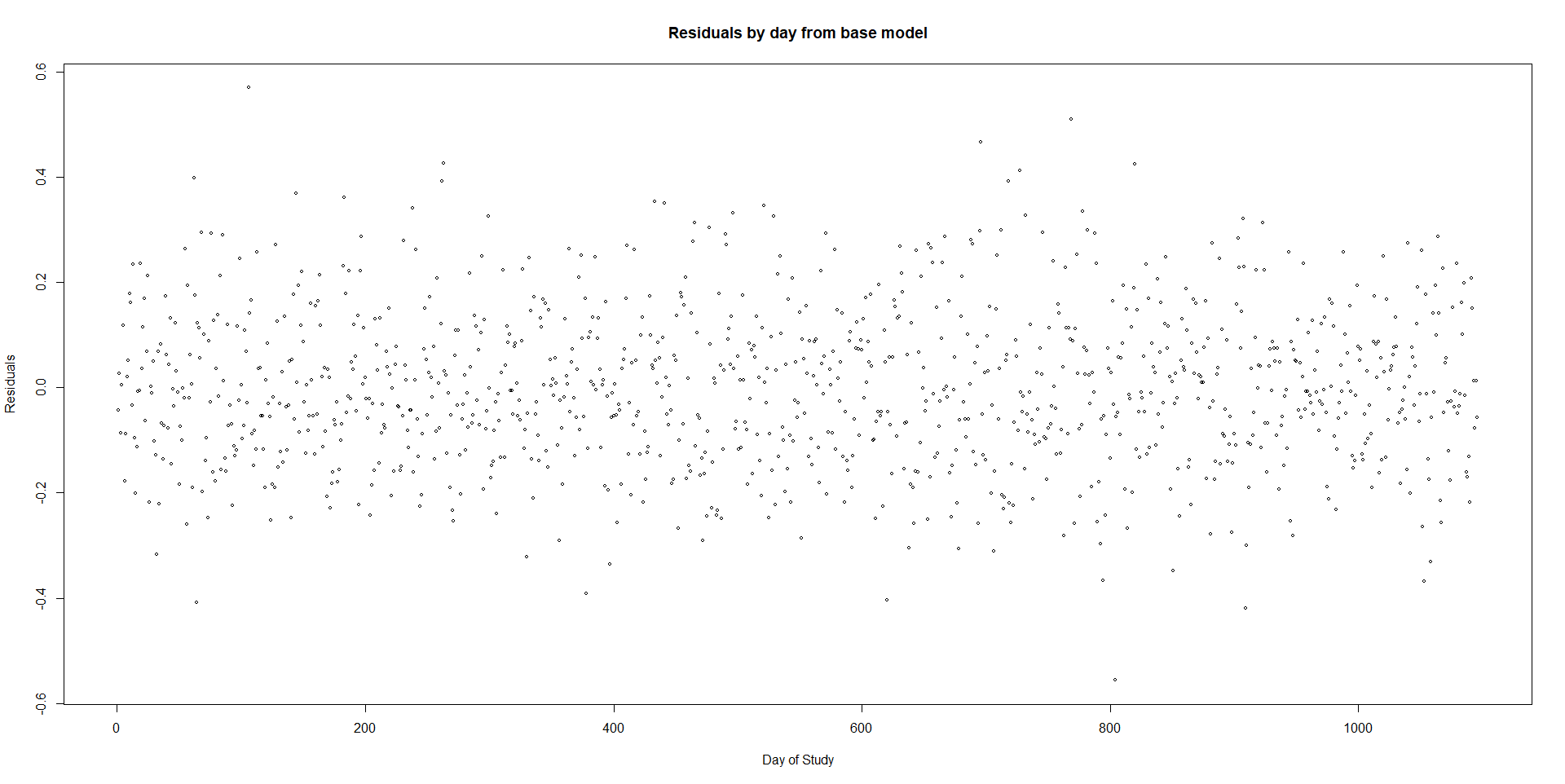

Supplement: S1 Fig — (DOCX) [file pone.0229258.s001.docx]
