# Supplementary material for: Heavy precipitation, drinking water source, and acute gastrointestinal illness in Philadelphia, 2015-2017
Source: PLoS One. 2020 Feb 24;15(2):e0229258. doi: 10.1371/journal.pone.0229258 (PMC7039462; doi:10.1371/journal.pone.0229258)
Supplement: S1 Table — (DOCX) [file pone.0229258.s002.docx]

**S1 Table. Association of daily precipitation and streamflow variables with AGI incidence, comparing AGI at exposure values >95^th^ percentile to ≤median (first RR peak after lag 0 shown), in crude model, primary model, and sensitivity analyses**

|  | Exposure – Precipitation at  Philadelphia International Airport | |  | Exposure – Precipitation within  combined 50-mile watershed^a^ | |  | Exposure – Combined streamflow^b^ | |
| --- | --- | --- | --- | --- | --- | --- | --- | --- |
| Model^c^ | Lag with  first peak  (days) | RR (95% CI) |  | Lag with  first peak  (days) | RR (95% CI) |  | Lag with  first peak  (days) | RR (95% CI) |
| All AGI |  |  |  |  |  |  |  |  |
| Crude model (no covariates), exposure >95p | Lag 10 | 1.013 (0.986, 1.040) |  | NA | No peak |  | Lag 0 | 1.029 (1.000, 1.058) |
| Primary model, mid-level precipitation  (>50p to ≤95p) | Lag 12 | 1.005 (0.998, 1.013) |  | Lag 10 | 1.002 (0.991, 1.014) |  | Lag 9 | 1.003 (0.997, 1.008) |
| Primary model, heavy precipitation (>95p) | Lag 10 | 1.014 (0.991, 1.038) |  | Lag 17 | 1.020 (0.995, 1.046) |  | Lag 9 | 1.015 (1.004, 1.027) |
| Sensitivity analysis of exposure >90p | Lag 15 | 1.003 (0.987, 1.019) |  | Lag 7 | 1.004 (0.987, 1.021) |  | Lag 9 | 1.016 (1.007, 1.024) |
| Sensitivity analysis of exposure >99p | NA | RRs<1 |  | Lag 17 | 1.023 (0.979, 1.069) |  | Lag 17 | 1.024 (1.001, 1.048) |
| Sensitivity analysis of day-of-year DF=14 | Lag 10 | 1.008 (0.985, 1.032) |  | Lag 18 | 1.012 (0.987, 1.039) |  | Lag 9 | 1.025 (1.012, 1.037) |
| Sensitivity analysis of day-of-year DF=4 | Lag 9 | 1.020 (0.997, 1.042) |  | Lag 13 | 1.008 (0.987, 1.031) |  | Lag 9 | 1.020 (1.008, 1.032) |
| Sensitivity analysis of temperature DF=6 | Lag 10 | 1.020 (0.995, 1.045) |  | Lag 11 | 1.024 (0.997, 1.052) |  | Lag 9 | 1.018 (1.005, 1.031) |
| Sensitivity analysis of temperature DF=2 | Lag 11 | 1.014 (0.992, 1.038) |  | Lag 16 | 1.037 (1.013, 1.060) |  | Lag 9 | 1.012 (1.001, 1.024) |
|  |  |  |  |  |  |  |  |  |
| Spring Season |  |  |  |  |  |  |  |  |
| Crude model (no covariates), exposure >95p | NA | No peak |  | Lag 9 | 1.056 (0.992, 1.124) |  | NA | No peak |
| Primary model, mid-level precipitation  (>50p to ≤95p) | NA | No peak |  | NA | No peak |  | Lag 9 | 1.110 (0.894, 1.379) |
| Primary model, heavy precipitation (>95p) | Lag 16 | 2.024 (1.163, 3.523) |  | Lag 11 | 5.839 (2.600, 13.11) |  | Lag 8 | 1.402 (0.924, 2.127) |
| Sensitivity analysis of exposure >90p | Lag 8 | 1.129 (0.822, 1.551) |  | Lag 10 | 1.627 (1.152, 2.298) |  | Lag 9 | 1.430 (0.959, 2.131) |
| Sensitivity analysis of exposure >99p | NA | No peak |  | Lag 13 | 7.575 (1.824, 31.45) |  | NA | No observations >99p |
| Sensitivity analysis of day-of-year DF=14 | Lag 16 | 1.645 (0.814, 3.326) |  | Lag 12 | 4.980 (2.105, 11.78) |  | Lag 8 | 1.574 (0.945, 2.620) |
| Sensitivity analysis of day-of-year DF=4 | Lag 16 | 1.580 (0.976, 2.559) |  | Lag 13 | 4.815 (2.261, 10.25) |  | NA | No peak |
| Sensitivity analysis of temperature DF=6 | Lag 16 | 1.580 (0.820, 3.045) |  | Lag 12 | 5.649 (2.263, 14.10) |  | NA | No peak |
| Sensitivity analysis of temperature DF=2 | Lag 17 | 1.872 (1.099, 3.190) |  | Lag 13 | 3.986 (1.889, 8.412) |  | Lag 8 | 1.230 (0.910, 1.661) |

AGI=acute gastrointestinal illness; RR=relative rate; CI=confidence interval; NA=not applicable due to no peak, RRs<1, or model did not converge; DF=degrees of freedom; p=percentile

^a^Average of daily mean precipitation within Delaware and Schuylkill River watersheds

^b^Average of Delaware River and Schuylkill River daily mean streamflow values

^c^All models except crude models include covariates for temperature, day-of-week, holidays, season, and temporal trends (natural spline variables for consecutive day of the study and day of the calendar year)
