# Supplementary material for: Heavy precipitation, drinking water source, and acute gastrointestinal illness in Philadelphia, 2015-2017
Source: PLoS One. 2020 Feb 24;15(2):e0229258. doi: 10.1371/journal.pone.0229258 (PMC7039462; doi:10.1371/journal.pone.0229258)
Supplement: S2 Table — (DOCX) [file pone.0229258.s003.docx]

**S2 Table.** **Association of daily precipitation and streamflow variables with AGI incidence, comparing AGI at exposure values >95^th^ percentile to ≤median (first RR peak after lag 0 shown) in models with- and without adjustment for 4-week cumulative precipitation**

|  | Exposure – Precipitation at  Philadelphia International Airport | | |  | Exposure – Precipitation within combined  50-mile watershed^a^ | | |  | Exposure – Combined streamflow^b^ | | |
| --- | --- | --- | --- | --- | --- | --- | --- | --- | --- | --- | --- |
| Model^c^ | QAIC | Lag with  first peak  (days) | RR (95% CI) |  | QAIC | Lag with  first peak  (days) | RR (95% CI) |  | QAIC | Lag with  first peak  (days) | RR (95% CI) |
| Overall |  |  |  |  |  |  |  |  |  |  |  |
| All AGI | 2946 | Lag 10 | 1.014 (0.991, 1.038) |  | 2943 | Lag 17 | 1.020 (0.995, 1.046) |  | 2917 | Lag 9 | 1.015 (1.004, 1.027) |
| All AGI, adj cumppt^d^ | 2979 | Lag 10 | 1.022 (0.983, 1.062) |  | 2966 | Lag 18 | 1.040 (1.002, 1.080) |  | 2941 | Lag 10 | 1.013 (1.001, 1.026) |
| Diarrhea | 1875 | Lag 9 | 1.043 (1.004, 1.082) |  | 1853 | Lag 18 | 1.049 (1.008, 1.092) |  | 1868 | Lag 9 | 1.017 (0.999, 1.035) |
| Diarrhea, adj cumppt | 1901 | Lag 9 | 1.057 (0.993, 1.124) |  | 1880 | Lag 18 | 1.077 (1.014, 1.144) |  | 1885 | Lag 9 | 1.012 (0.992, 1.032) |
| Vomiting | 2690 | Lag 11 | 1.006 (0.983, 1.030) |  | 2694 | Lag 14 | 1.011 (0.987 ,1.035) |  | 2658 | Lag 10 | 1.018 (1.007, 1.030) |
| Vomiting, adj cumppt | 2717 | Lag 9 | 1.016 (0.977, 1.058) |  | 2715 | Lag 17 | 1.027 (0.988, 1.068) |  | 2679 | Lag 10 | 1.016 (1.003, 1.029) |
| By Season |  |  |  |  |  |  |  |  |  |  |  |
| Winter | 978 | Lag 8 | 1.177 (0.866, 1.601) |  | 978 | Lag 9 | 1.176 (0.849, 1.630) |  | 967 | Lag 12 | 1.101 (0.824, 1.470) |
| Winter, adj cumppt | 1004 | NA | No peak |  | 1008 | NA | No peak |  | 992 | NA | No peak |
| Spring | 701 | Lag 16 | 2.024 (1.163, 3.523) |  | 668 | Lag 11 | 5.839 (2.600, 13.11) |  | 711 | Lag 8 | 1.402 (0.924, 2.127) |
| Spring, adj cumppt | 685 | Lag 14 | 1.908 (0.947, 3.846) |  | 665 | Lag 11 | 3.808 (1.297, 11.18) |  | 702 | NA | No peak |
| Spring – Diarrhea | 459 | Lag 15 | 3.774 (1.590, 8.960) |  | 463 | Lag 11 | 8.441 (2.308, 30.86) |  | 482 | Lag 9 | 1.833 (0.907, 3.701) |
| Spring - Diarrhea, adj cumppt | 484 | Lag 12 | 5.129 (1.505, 17.48) |  | 444 | Lag 11 | 10.31 (1.908, 55.76) |  | 483 | NA | No peak |
| Spring – Vomiting | 641 | Lag 16 | 1.824 (1.030, 3.228) |  | 603 | Lag 11 | 5.962 (2.601, 13.67) |  | 641 | Lag 8 | 1.391 (0.908, 2.130) |
| Spring - Vomiting, adj cumppt | 623 | Lag 14 | 1.653 (0.804, 3.400) |  | 607 | Lag 11 | 3.668 (1.211, 11.12) |  | 640 | NA | No peak |
| Summer | 540 | NA | No peak |  | 521 | NA | RRs<1 |  | 520 | NA | No peak |
| Summer, adj cumppt | 523 | Lag 14 | 1.248 (0.625, 2.492) |  | 516 | NA | No peak |  | 528 | NA | No peak |
| Fall | 652 | NA | No peak |  | 619 | NA | No peak |  | NA | NA | No observations >95p |
| Fall, adj cumppt | 660 | NA | No peak |  | 655 | NA | Peak at long lag |  | NA | NA | No observations >95p |

AGI=acute gastrointestinal illness; RR=relative risk; CI=confidence interval; NA=not applicable due to no peak, RRs<1, or model did not converge

^a^Average of daily mean precipitation within Delaware and Schuylkill River watersheds

^b^Average of Delaware River and Schuylkill River daily mean streamflow values

^c^All estimates are adjusted for temperature, day-of-week, holidays, and temporal trends (natural spline variables for consecutive day of the study and day of the calendar year). Non-season specific estimates are also adjusted for season using indicator variables.

^d^Additional adjustment for 4-week cumulative precipitation
